# Supplementary material for: Intrapapillary capillary loop classification in magnification endoscopy: open dataset and baseline methodology
Source: Int J Comput Assist Radiol Surg. 2020 Mar 12;15(4):651–9. doi: 10.1007/s11548-020-02127-w (PMC7142046; doi:10.1007/s11548-020-02127-w)
Supplement: Supplementary file 1 — Supplementary material 1 (pdf 234 KB) [file 11548_2020_2127_MOESM1_ESM.pdf]

**Int. J. Comput. Assist. Radiol. Surg. manuscript No.**
(will be inserted by the editor)

# Intrapapillary Capillary Loop Classification in Magnification Endoscopy: Open Dataset and Baseline Methodology

## Supplementary Material

**Luis C. García-Peraza-Herrera · Martin Everson · Laurence Lovat · Hsiu-Po Wang · Wen Lun Wang · Rehan Haidry · Danail Stoyanov · Sébastien Ourselin · Tom Vercauteren**

L. C. Garcia-Peraza-Herrera
Department of Medical Physics and Biomedical Engineering, UCL, London, UK E-mail: luis.herrera.14@ucl.ac.uk

M. Everson, L. Lovat, R. Haidry
Division of Surgery & Interventional Science, UCL, Department of Gastroenterology; University College Hospital NHS Foundation Trust, London, UK

H. Wang
Department of Internal Medicine, National Taiwan University, Taipei, Taiwan

W. L. Wang
Department of Internal Medicine, E-Da Hospital/I-Shou University, Kaohsiung, Taiwan

D. Stoyanov
Wellcome / EPSRC Centre for Interventional and Surgical Sciences, UCL, London, UK

L. C. Garcia-Peraza-Herrera, S. Ourselin, T. Vercauteren
School of Biomedical Engineering & Imaging Science, KCL, UK

## A Number of patients and frames per fold

The number of patients per fold is shown in appendix table A1. Similarly, the number of frames is shown in appendix table A2.

**Table A1** Number of patients per fold (80% training, 10% validation, 10% testing).

| Dataset | **Fold** 1 | **Fold** 2 | **Fold** 3 | **Fold** 4 | **Fold** 5 |
|---|---|---|---|---|---|
| Training (normal) | 36 | 35 | 39 | 36 | 36 |
| Training (abnormal) | 55 | 56 | 52 | 55 | 55 |
| Training (normal + abnormal) | 91 | 91 | 91 | 91 | 91 |
| Validation (normal) | 5 | 6 | 2 | 5 | 6 |
| Validation (abnormal) | 6 | 5 | 9 | 6 | 5 |
| Validation (normal + abnormal) | 11 | 11 | 11 | 11 | 11 |
| Testing (normal) | 4 | 4 | 4 | 4 | 3 |
| Testing (abnormal) | 8 | 8 | 8 | 8 | 9 |
| Testing (normal + abnormal) | 12 | 12 | 12 | 12 | 12 |

**Table A2** Number of frames per fold.

| Dataset | **Fold** 1 | **Fold** 2 | **Fold** 3 | **Fold** 4 | **Fold** 5 |
|---|---|---|---|---|---|
| Training (normal) | 22857 | 22883 | 23467 | 20504 | 24671 |
| Training (abnormal) | 33160 | 31959 | 33024 | 34905 | 28233 |
| Training (normal + abnormal) | 56017 | 54842 | 56491 | 55409 | 52904 |
| Validation (normal) | 2667 | 3534 | 1649 | 4793 | 2781 |
| Validation (abnormal) | 2522 | 3883 | 4334 | 1500 | 3237 |
| Validation (normal + abnormal) | 5189 | 7417 | 5983 | 6293 | 6018 |
| Testing (normal) | 2555 | 1662 | 2963 | 2782 | 627 |
| Testing (abnormal) | 3981 | 3821 | 2305 | 3258 | 8193 |
| Testing (normal + abnormal) | 6536 | 5483 | 5268 | 6040 | 8820 |

## B Qualitative classification results and cases of patient failure

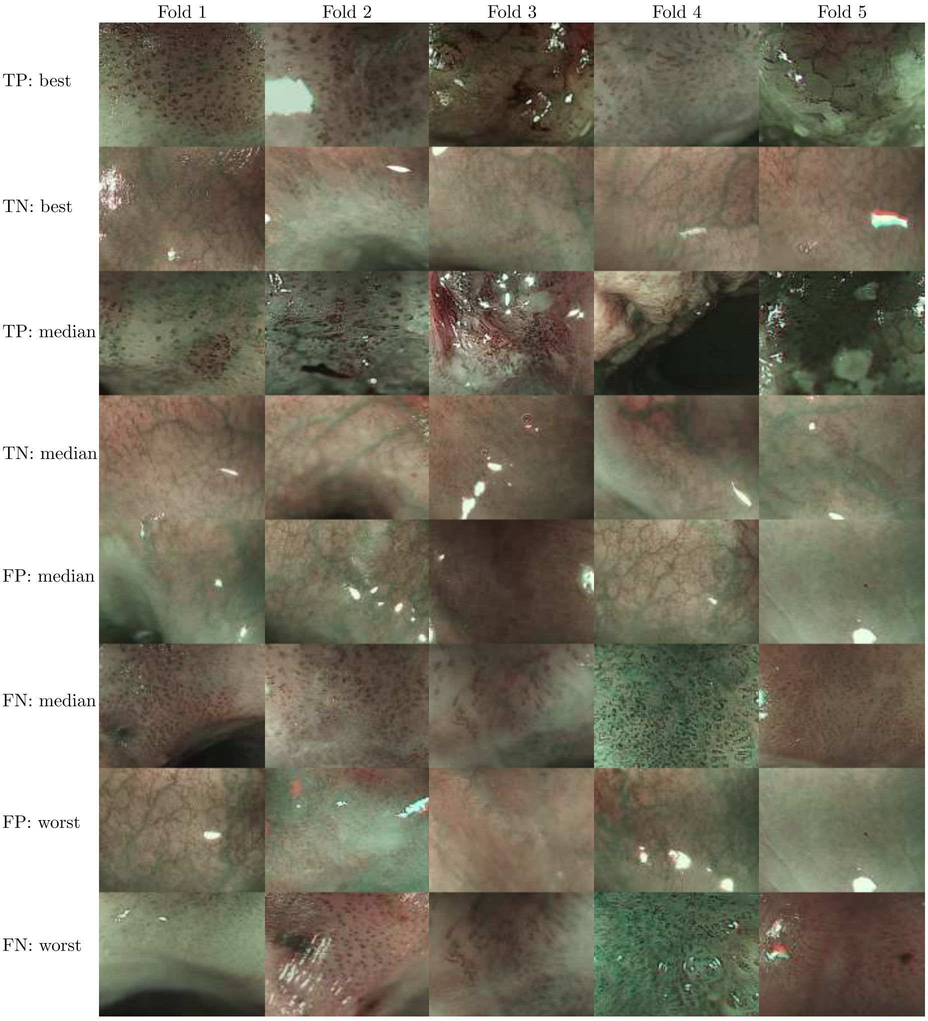


**Figure A1** Qualitative results for ResNet-18-CAM-DS on frame classification over the testing set of each fold. TP, TN, FP, FN stand for true positives, true negatives, false positives and false negatives respectively. *Best* TP refers to the abnormal image with the highest estimated probability of being abnormal. Analogously, the *best* TN represents the image with lowest estimated probability. Median and worst cases are estimated in a similar fashion. The FP median and worst case of fold 5 are the same image because in the testing set of this fold there is only this false positive image.

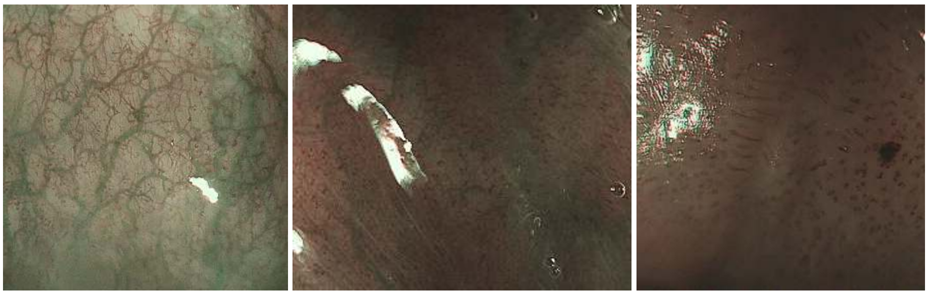

**Figure A2** Representative frames of cases of patient failure (i.e. when the average estimated class for the whole patient clip is wrong). Patient 158 (normal IPCL, left), 143 (normal IPCL, center), 66 (right, abnormal IPCL). ResNet-18 failed on all of them. ResNet-18-CAM only on 143 and 66. ResNet-18-CAM-DS failed only on case 158.

## C ROC result for ResNet-18-CAM-DS

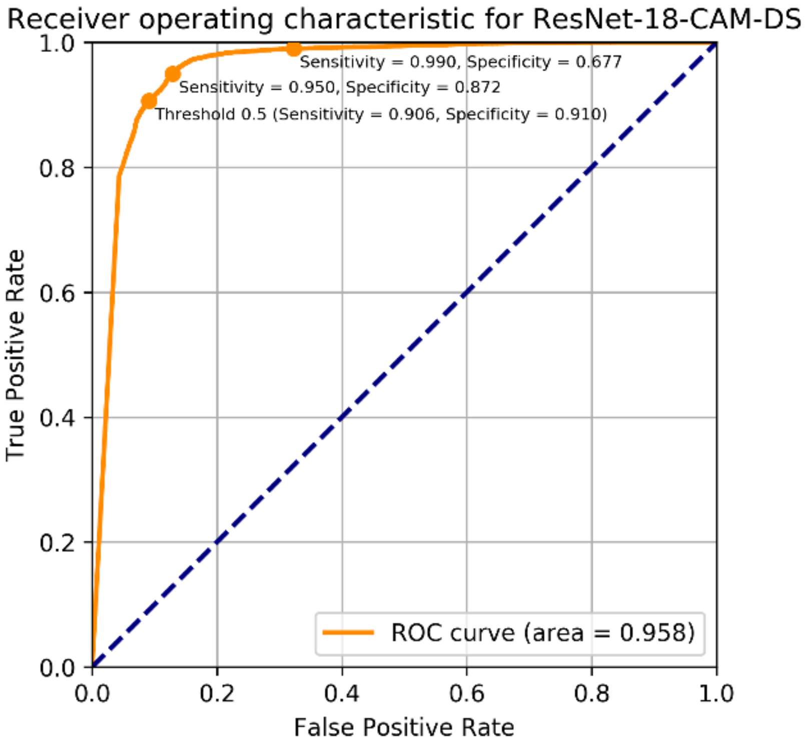


**Figure A3** ROC curve based on the ResNet-18-CAM-DS predictions. Frames belonging to the testing set of all the folds were considered (although predictions for each fold were estimated with the network trained for such fold). As our system is intended to be used as a CADe, operating points at a sensitivity of 95 percent and 99 percent are shown for illustrative purposes.
